# About the influence of environmental factors on the persistence of DNA — a long-term study

**DOI:** 10.1007/s00414-022-02800-6

**Published:** 2022-02-23

**Authors:** Micaela Poetsch, Philipp Markwerth, Helen Konrad, Thomas Bajanowski, Janine Helmus

**Affiliations:** Institute of Legal Medicine, University Hospital Essen, Hufelandstr. 55, 45122 Essen, Germany

**Keywords:** Persistence of DNA, Indoor, Outdoor, Weather, Environment, Temperature, STR analysis, Low-copy number DNA

## Abstract

DNA persistence and DNA transfer are important features in the assessment of a crime scene. The question how long DNA may persist at a certain location is similarly important as the one how the DNA has been transferred to this location. Depending on the source of the DNA as well as the conditions at the crime scene, the answer to this question is quite difficult. In this study, persistence of DNA from epithelial abrasions, blood cells, and saliva cells in indoor and outdoor scenarios has been investigated with regard to exposure time and exposure conditions including sunlight, temperature, and humidity in summer and winter scenarios. Overall, we generated 338 epithelial samples, 572 blood samples, and 572 saliva samples. A complete profile of the cell/DNA donor after exposure could be obtained in 47%, 65%, and 58% of epithelial abrasions, blood samples, and saliva samples, respectively. Regarding blood samples, there were no differences between supporting materials cloth and plastic; however, the percentage of complete profiles was higher for saliva samples on plastic and for epithelial samples on cloth. In indoor scenarios, complete profiles could be recovered from nearly all blood and saliva samples up to 9 months, whereas the amount of epithelial complete profiles already started to decline after 3 months. In outdoor scenarios, we observed a tipping point at an exposure time of 3 months. Blood and saliva samples collected after this period displayed complete profiles in less than 25% of samples. After 12 months, no outdoor sample showed a complete profile. The results of this study facilitate decisions on the relevance of recovered DNA from crime scenes.

**Supplementary Information:**

The online version contains supplementary material available at 10.1007/s00414-022-02800-6.

## Introduction

The great progress in forensic DNA analysis has led to DNA testing being conducted regularly and on a large scale during police investigations [1], since it is now possible to create DNA profiles from even the smallest amounts of DNA secured at a crime scene [2]. However, the impact of these DNA traces has to be evaluated carefully [3–5]. Here, two main features should be considered: first, in which way has the DNA been transferred to the location or item and second, how long could the DNA have persisted at this location/on this item before sampling. Depending on the source of the DNA as well as the conditions at the crime scene, the answer to the second question is quite difficult. While several studies deal with the possibilities of DNA transfer [e.g., 6–9], studies on the persistence of DNA are still rare and have mostly been conducted under laboratory conditions [10–13]. Investigations of outdoor scenarios are mainly limited to restricted conditions [14–19]. Especially, the influence of weather on DNA stability is a very rare topic. A recent study run in Singapore under tropical conditions showed that DNA persistence on items left outdoors showed a great variation, mostly dependent on the amount of rain [20]. Therefore, we investigated DNA samples from epithelial abrasions, blood cells, and saliva cells in indoor and outdoor scenarios under humid temperate climate conditions.

## Material and methods

### Samples

The study comprised blood, saliva, and epithelial abrasions from two individuals of different age (35 years, 53 years), without any known skin disease to exclude an influence of the shedder status as described by Kamphausen et al. [21]. Samples were collected in 2019 and 2020 in the Institute of Legal Medicine, University Hospital Essen, Germany.

### Compliance with ethical standards

All samples were obtained after informed consent and with approval of the Medical Ethics Committee at the University of Duisburg-Essen in accordance with the Declaration of Helsinki and national laws (ethic vote number: 09–3950, 20–9523-BO).

### Artificial scenarios

Several different scenarios were set up (Table 1); in each one we used 20 µl and 100 µl of blood and saliva, directly pipetted on cloth and plastic, as well as neck abrasions on cloth and plastic, gained by rubbing cloth or plastic over the neck for approximately 5 s with medium pressure (Figure S1). Application areas for blood and saliva were marked to facilitate retrieval of cells/DNA. Exposure times started with 5 days and went up to 12 months (Table 1). For each scenario, four blood samples, four saliva samples, and two neck abrasions were analyzed. Every scenario with an exposure time of at least 2 weeks was executed twice for every material/amount. Moreover, several scenarios with exposure times of at least 1 month were carried out in summer and winter months (see Table S1 for weather conditions in summer and winter as well as definition of summer and winter for this study). After the appropriate period, cloth and plastic pieces were collected and dried in a DNA-free environment. Negative controls of cloth and plastic were investigated prior to execution of scenarios. Overall, we generated 338 epithelial samples, 572 blood samples, and 572 saliva samples.

**Table 1:** Artificial scenarios

Time period	Indoor, room temperature		Outdoor, environmental temperature						Number of samples
	In the dark	Under sunlight^1^	Dark and dry, on soil	Light and dry, on soil	Light and exposed, on soil	Dark and dry, on polystyrene	Light and dry, on polystyrene	Light and exposed, on cloth	
5 days^4^; s	x	x	x	x	x	x	x	x	82
1 week^3^; s	x	x	x	x	x	x	x	x	80
2 weeks^2^; s	x	x	x	x	x	x	x	x	160
3 weeks^2^; s			x	x	x				120
1 month^2^; s, w	x	x	x	x	x	x	x	x	320
3 months^2^; s, w	x	x	x	x	x	x	x	x	320
6 months^2^; s, w	x	x	x			x	x		200
9 months^2^	x	x	x			x	x		100
12 months^2^	x	x	x			x	x		100

*x* scenario was executed, *s* summer months, *w* winter months (see Table S1 for definition).

^1^Samples were stored on a windowsill facing southwest to increase the amount of sunlight as much as possible.

^2^For each of these scenarios, four blood samples (20 µl and 100 µl, on cloth and plastic), four saliva samples (20 µl and 100 µl, on cloth and plastic), and two neck abrasions (on cloth and plastic) were analyzed. Every scenario was executed twice.

^3^For these scenarios, four blood samples (20 µl and 100 µl, on cloth and plastic), four saliva samples (20 µl and 100 µl, on cloth and plastic), and two neck abrasions (on cloth and plastic) were analyzed without repetition.

^4^For these scenarios, four blood samples (20 µl and 100 µl, on cloth and plastic), four saliva samples (20 µl and 100 µl, on cloth and plastic), and two neck abrasions (on cloth and plastic) were analyzed. A repetition war only executed for neck abrasions under the conditions, “light and exposed, on cloth”.

### DNA extraction, quantification, amplification, and electrophoresis

Collecting of DNA from plastic surfaces as well as cotton clothes was done as described before [22]. DNA extraction was performed using DNA IQ Casework Pro Kit and Casework Extraction Kit in the Maxwell 16® instrument according to the manufacturer’s instructions (Promega, Mannheim, Germany), resulting in an extraction volume of 50 µl. DNA concentration of samples was established by Real-time PCR using the PowerQuant™ System (Promega) according to the manufacturer’s instructions providing a reproducible and reliable detection threshold at least down to 25 pg DNA [23]. Using 2 µl DNA-containing solutions each sample was analyzed in duplicates. DNA amplification with multiplex PCR Kit Powerplex® ESX17fast, evaluation on an ABI3500 Genetic Analyzer (Applied Biosystems) with GeneMapper® ID-X Software, and assessment of results regarding complete profiles were done as described before [9]. In short, a result was regarded as a complete profile, if in every evaluable locus every allele of the cell donor was found.

## Results and discussion

### Reliability of data and DNA concentrations

All negative controls showed no profiles; DNA amount was either negative (undetermined according to PowerQuant System) or below 0.0005 ng/µl.

First, we established expected DNA yields for our samples by analyzing 16 different samples for each chosen amount of blood and saliva as well neck abrasions (Table S2). Here, 20 µl and 100 µl blood resulted in 152–928 ng DNA (mean 465 ng) and 693–3898 ng DNA (mean 1438 ng), respectively. DNA yield of saliva samples was slightly lower, since 20 µl and 100 µl saliva contained 190–667 ng DNA (mean 381 ng) and 262–1617 ng DNA (mean 735 ng), respectively. As expected, recovery of DNA from neck abrasions was less successful with 17.7–44.5 ng (mean 28 ng), but still sufficient to create a complete profile of the donor.

DNA concentrations of samples from the different scenarios measured by Real-time PCR varied widely between the different experimental setups as well as different cell origins (Table S3), but corresponded to the results of the STR analysis (samples with a DNA concentration below 0.0005 ng/µl demonstrated no profiles). As expected, DNA concentrations of samples from experiments with saliva or blood were higher than regarding experiments with skin cells. Loss of DNA over exposure time occurred in different amounts between different scenarios. After 3 months indoors, nearly 80% and 70% of DNA from blood and saliva were lost, respectively (Table S3). Similar values could be obtained in outdoor scenarios with a mean loss of 92% (blood) and 97% (saliva), although with a greater range (0–47% and 0–9% recovery rate for blood and saliva, respectively). Regarding epithelial abrasions, DNA amount after 1 month varied between 1.7 ng and 25.3 ng in the indoor scenarios, whereas 0–11.5 ng DNA could be found in epithelial abrasions regarding outdoor scenarios. These values are lower than those reported by Lee et al. [20] with 28–137 ng DNA in keratinocyte suspensions in indoor scenario after 4 weeks, but higher than those detected on wristbands (0–0.39 ng) [20], the last setup being much more comparable to our scenarios. After 12 months, we lost 100% of DNA in nearly every outdoor scenario, which is in line with the results from Lee et al. [20].

### STR amplification

Looking at the results with no regard to the scenario, complete profiles could be demonstrated in 861 samples (58% of all samples). Only 177 samples (12%) showed a partial profile, distributed equally about different scenarios and cell origins. Since the amount of partial profiles in this study is rather low, we decided to concentrate on complete profiles only for all further evaluations. Moreover, although a partial profile may sometimes provide information for a possible assignment of a person to a DNA samples, it does not offer enough certainty to allow a biostatistical calculation in Germany [24, 25].

### Relevance of cell origin

Considering cellular origin, 373 blood samples (65% of blood samples), 330 saliva samples (58% of saliva samples), and 158 epithelial samples (47% of epithelial samples) showed a complete profile (Table 2). These differences were not surprising, since fresh blood contains about 4000–10,000 leukocytes/µl [26], while other studies have shown that saliva samples can be expected to contain about 3000 cells/ml, with highly variable composition [27, 28]. Regarding epithelial samples, it is known that the majority of the cells are nuclei-free [29].

**Table 2:** Distribution of the amount of complete profiles with regard to cell origin and supporting material

	Blood samples (*n* = 572)*	Saliva samples (*n* = 572)*	Epithelial samples (*n* = 338)*
Complete profiles	373 (65%)	330 (58%)	158 (47%)
Complete profiles on plastic	182 ≙ 49% of CP ≙ 64% of blood samples on plastic	177 ≙ 54% of CP ≙ 62% of saliva samples on plastic	69 ≙ 44% of CP ≙ 41% of epithelial samples on plastic
Complete profiles on cloth	191 ≙ 51% of CP ≙ 67% of blood samples on cloth	153 ≙ 46% of CP ≙ 53% of saliva samples on cloth	89 ≙ 56% of CP ≙ 53% of epithelial samples on cloth

*Half of samples were on plastic, half on cloth.

A higher resilience of saliva samples has already been demonstrated [22] and may be due to cell composition (salivary cells, inflammatory cells) or the presence of glycoproteins [30] which enhance the adhesion of cells and DNA to the surface [31].

### Influence of supporting material

There were no differences in the number of complete profiles between samples on plastic and samples on cloth (428 and 433 complete profiles, respectively). However, the results did vary, if the origin of the cells was also considered (Table 2). On cloth, epithelial samples are more prone to conserve enough DNA for a complete profile than on plastic, a phenomenon which has been described before [31, 32]. Moreover, epithelial cells detach more easily from smooth surfaces and got lost as shown by Goray et al. [33]. In contrast, it is more probable to detect a complete profile from saliva samples on plastic than on cloth, which again may be due to the presence of glycoproteins that enhance the adhesion of cells and DNA to the surface [30].

### Indoor scenarios

**Fig. 1:**
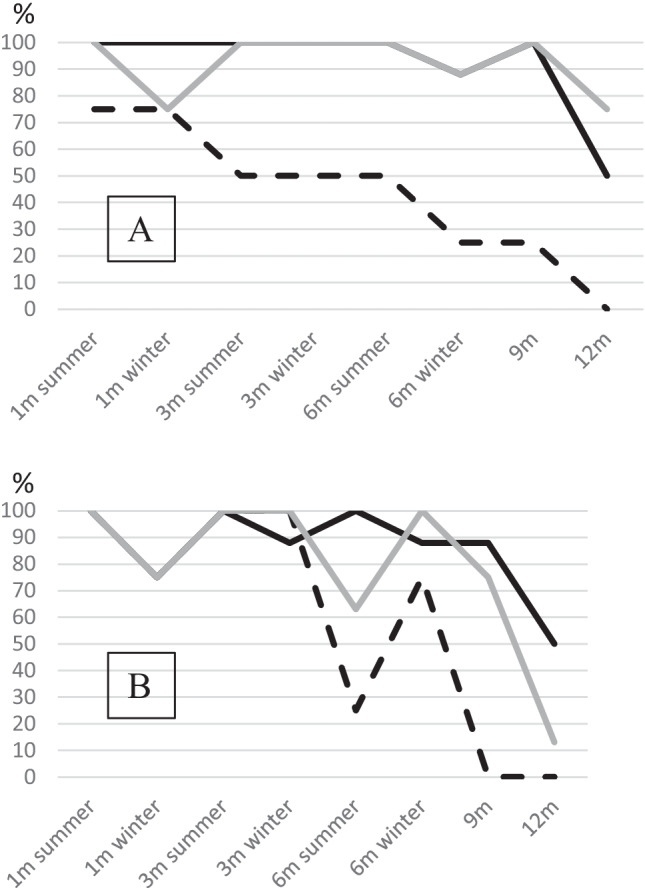
Graphical display of the percentage of samples with complete profile with regard to the different indoor scenarios and exposure time (starting with 1 month). **A** In the dark and **B** under sunlight. Black lines represent blood samples, grey lines represent saliva samples, and interrupted lines stand for epithelial samples

For an exposure period of up to 9 months, nearly all blood and saliva samples stored in the dark resulted in complete profiles as expected (Fig. 1), since especially blood samples collected on paper or cloth are routinely stored for much longer time frames. Rather surprisingly, only 50% of blood samples and 75% of saliva samples demonstrated all alleles of the responsible individual after an exposure of 12 months. This can possibly be explained by the use of plastic as supporting material as well as the rather low amount of 20 µl blood in some samples, since these samples showed the most allele losses. Moreover, we often observed a flaking of blood samples thus reducing the amount further. Regarding epithelial abrasions, the results are quite different (Fig. 1). After 3 months, only half of samples demonstrated a complete profile, after 12 months none, not even those in the dark. Here, not UV radiation as expected but another factor seems to be relevant. Possibly the different composition of the bacterial fauna on the skin compared to, for example, the bacterial fauna in saliva could have an influence [34].

The influence of sunlight was investigated in the second indoor scenario. Blood and saliva samples showed a high amount of complete profiles up to 9 months of storage (Fig. 1); however, only 13% of saliva samples demonstrated all expected alleles after 12 months. In blood samples, we found complete profiles in 50% of samples, same as in the dark scenario. Generally, the destructive effects of UV radiation on DNA are well known and used for removal of DNA from instruments and surfaces [35]. However, terrestrial UV light alone has already been shown to be an only minor contributor to DNA damage [36] as confirmed by the results of this study.

### Outdoor scenarios

The outdoor scenarios were divided in two main sections: the storage was done either on soil or on polystyrene/cloth to get some insights in the influence of chemical components of the soil as well as bacteria or small animals as insects or worms.

**Fig. 2:**
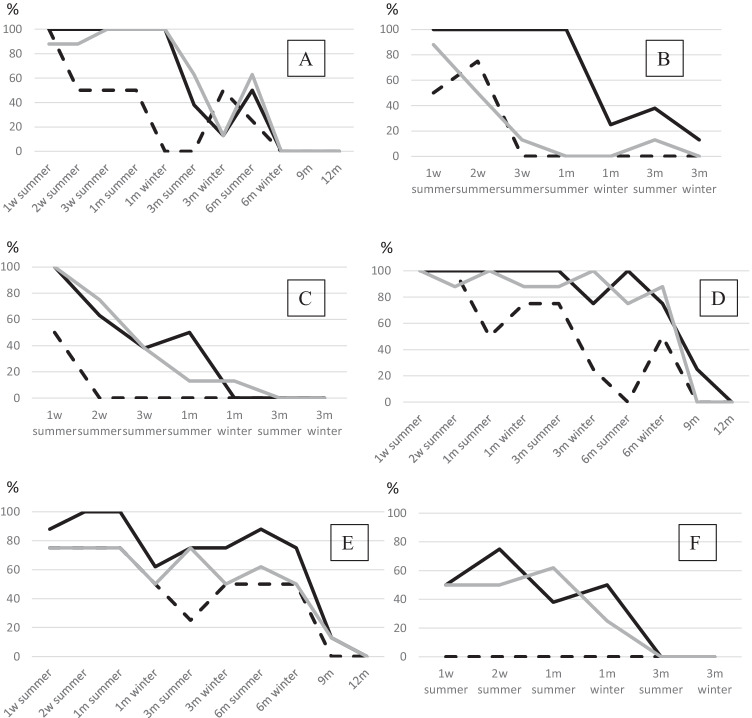
Graphical display of the percentage of samples with complete profiles with regard to the different outdoor scenarios and exposure times (starting with 1 week). **A** Dark and dry on soil, **B** light and dry on soil, **C** light and exposed on soil, **D** dark and dry on polystyrene, **E** light and dry on polystyrene, and **F** light and exposed on cloth. Black lines represent blood samples, grey lines represent saliva samples, and interrupted lines stand for epithelial samples

Not surprisingly, the portion of complete profiles in general was lower in outdoor than in indoor scenarios (Fig. 2). Especially on soil, an exposure time of 3 months proved to be a tipping point; after that, only few complete profiles could be found in blood and saliva samples (Fig. 2). This tipping point seems also be true for the unprotected blood and saliva samples on cloth (Fig. 2). Epithelial abrasions rendered no results as early as after 2 weeks (Fig. 2C, completely exposed), 3 weeks (Fig. 2B, with light), or 1 month (Fig. 2A, in the dark). In contrast, on polystyrene, complete profiles could be demonstrated for as long as 9 months in individual blood and saliva samples and as long as 6 months in epithelial abrasions (Fig. 2). While Lee et al. found a significant influence of the growth of fungus on the prevalence of DNA in the outdoor scenarios [20], direct contact with the bacterial flora of the soil seems to be the main influencing factor here. This is in line with another study investigating the stability of DNA in direct contact with soil [37]. A direct correlation between growth of fungus/bacteria with resulting loss of DNA to the amount of rainfall during the study period as described by Lee et al. [20] could not be observed in this study. This may be due to the difference in climate as well as regarding the amount of rainfall which is much lower in this study than in Singapore.

In general, samples from the summer scenarios demonstrated more complete profiles than those of winter scenarios, regardless of the source of DNA (Table S4). While 52% of all summer samples showed a complete profile, only 37% of winter samples had this result. The most striking differences could be found in blood samples, since in 61% of summer samples but only in 38% of winter samples all alleles of the responsible person were detected. Therefore, the influence of lower temperatures and — especially — a higher humidity seems to outrank that of sunlight. The influence of humidity on DNA persistence has already been discussed in several studies [19, 38, 39], whereby in this study especially milder temperatures and high humidity may have favored microbial colonization. Another study has also described this phenomenon [40]. Moreover, the much greater loss of DNA from any source on soil than on polystyrene also favors this explanation. In this context, it should be noted that in 2019 the summer was relatively dry.

## Conclusion

The results of this study demonstrate a rather distinctive time frame for a possible recovery of DNA from blood, saliva, and epithelial cells after exposure to sun and rain as well as high and low temperatures. This information could be helpful during police investigation and in court to evaluate the chances of successful DNA typing and to facilitate decisions on the relevance of recovered DNA from crime scenes.

## Supplementary Information

Below is the link to the electronic supplementary material.

Supplementary file1 (DOCX 1959 KB)

Supplementary file2 (DOCX 13 KB)

Supplementary file3 (DOCX 14 KB)

Supplementary file4 (XLSX 16 KB)

Supplementary file5 (DOCX 12.6 KB)
