# Supplementary material for: About the influence of environmental factors on the persistence of DNA — a long-term study
Source: Int J Legal Med. 2022 Feb 23;136(3):687–93. doi: 10.1007/s00414-022-02800-6 (PMC9005405; doi:10.1007/s00414-022-02800-6)
Supplement: Supplementary file 1 — Supplementary file1 (DOCX 1959 KB) [file 414_2022_2800_MOESM1_ESM.docx]

**Figure S1: Example pictures for the scenarios**


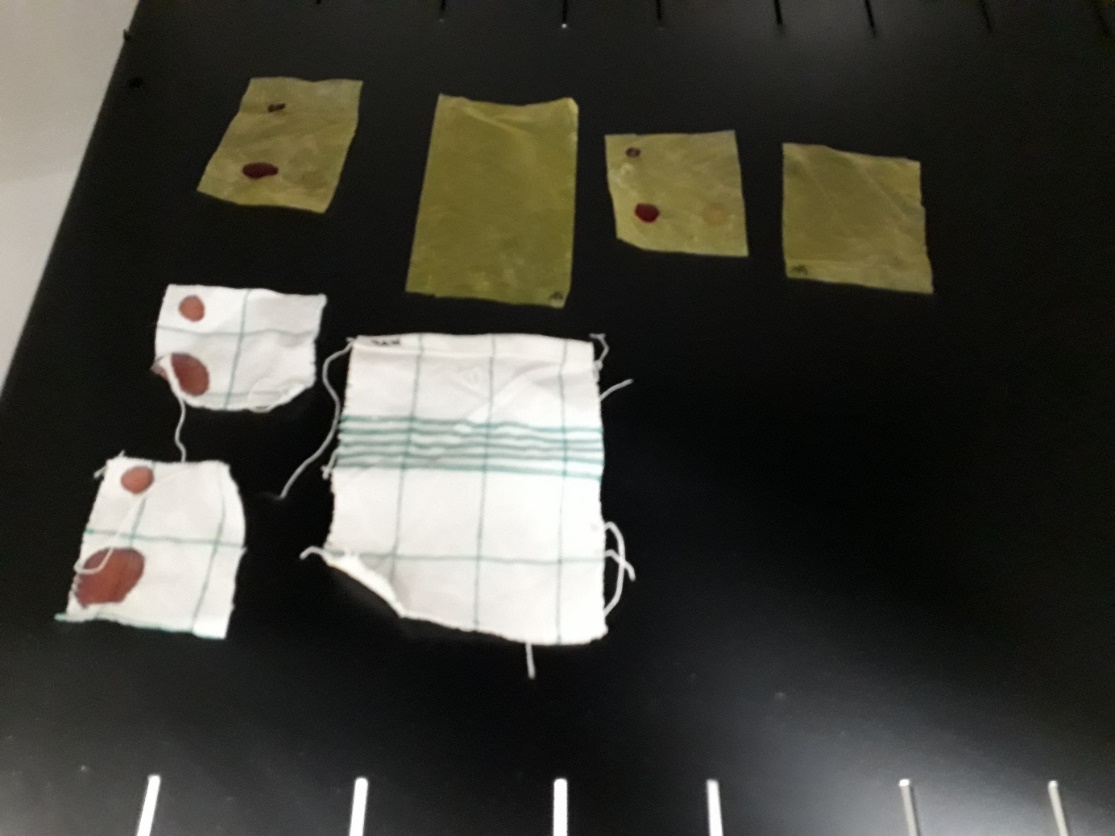


a: Set-up of a scenario


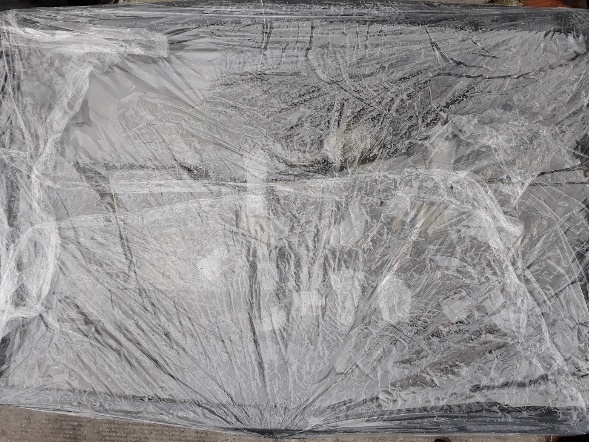

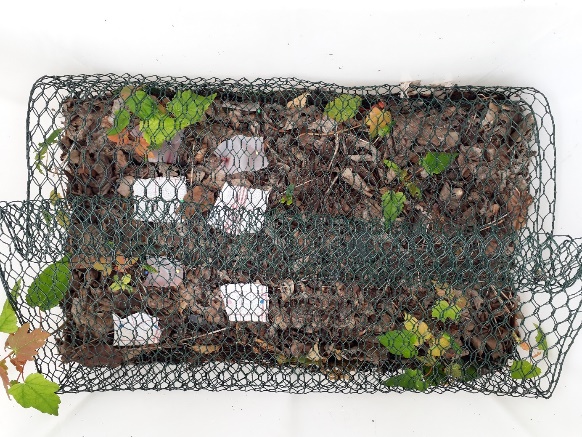


b: light and dry, on soil c: light and exposed, on soil


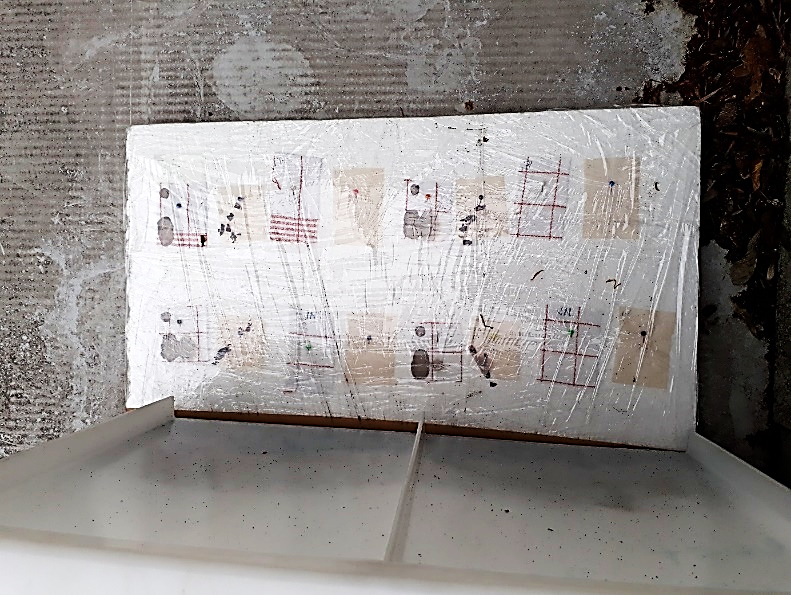

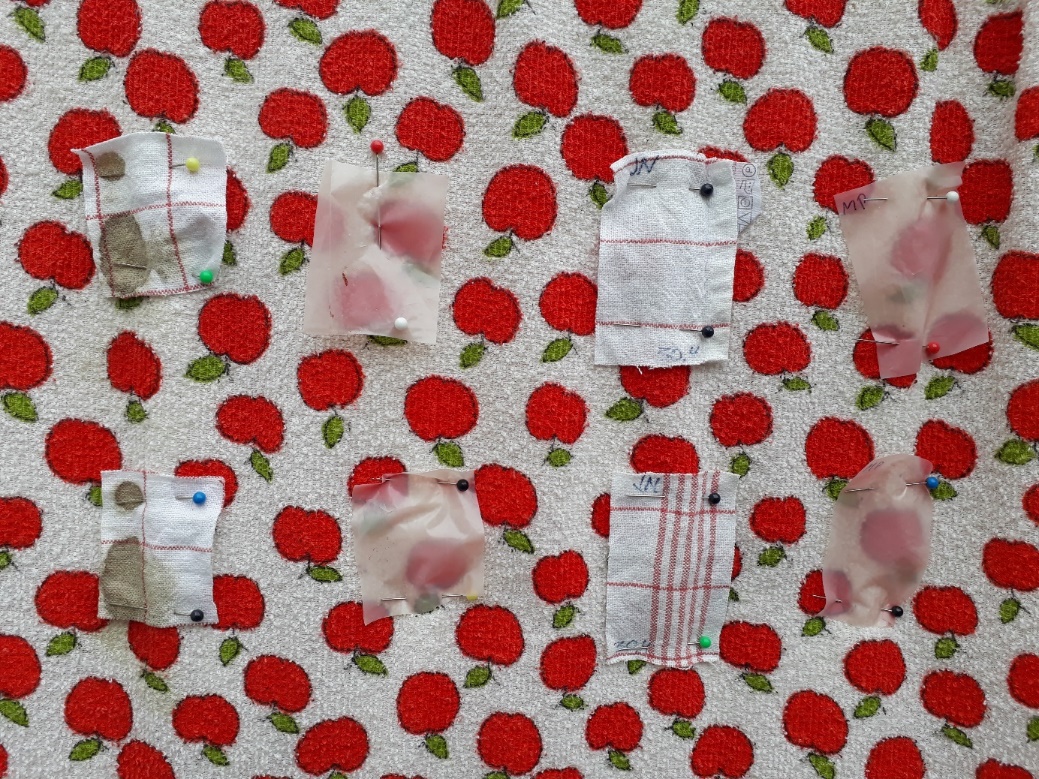


d: light and dry, on polystyrene e: light and exposed, on cloth
