# Supplementary material for: About the influence of environmental factors on the persistence of DNA — a long-term study
Source: Int J Legal Med. 2022 Feb 23;136(3):687–93. doi: 10.1007/s00414-022-02800-6 (PMC9005405; doi:10.1007/s00414-022-02800-6)
Supplement: Supplementary file 2 — Supplementary file2 (DOCX 13 KB) [file 414_2022_2800_MOESM2_ESM.docx]

**Table S1: Weather conditions for summer and winter scenarios**

|  |  | **Summer*** | **Winter*** |
| --- | --- | --- | --- |
| **Temperature** | Mean | 17.1 °C | 6.0 °C |
|  | Lowest | 7.2 °C | -4.5 °C |
|  | Highest | 35.1 °C | 17.4 °C |
| **Sunshine** |  | 878.4 hours | 334.1 hours |
| **Amount of rain** |  | 176.1 l/m² | 395.7 l/m² |
| **Rainy days** |  | 51 | 57 |

* summer: between April and September; winter: between October and March
