# Supplementary material for: About the influence of environmental factors on the persistence of DNA — a long-term study
Source: Int J Legal Med. 2022 Feb 23;136(3):687–93. doi: 10.1007/s00414-022-02800-6 (PMC9005405; doi:10.1007/s00414-022-02800-6)
Supplement: Supplementary file 3 — Supplementary file3 (DOCX 14 KB) [file 414_2022_2800_MOESM3_ESM.docx]

**Table S2: Total DNA amount in blood, saliva and neck abrasions (in ng)**

|  | 20 µl blood | 100 µl blood | 20 µl saliva | 100 µl saliva | Neck abrasion |
| --- | --- | --- | --- | --- | --- |
| Sample 1 | 242.5 | 1093 | 351.5 | 1616.5 | 31.04 |
| Sample 2 | 362.5 | 755.5 | 418.5 | 730.5 | 23.66 |
| Sample 3 | 151.5 | 1062.5 | 478 | 841.5 | 20.61 |
| Sample 4 | 755 | 1268.5 | 464 | 975.5 | 26.16 |
| Sample 5 | 465.5 | 2111 | 424 | 873 | 31.25 |
| Sample 6 | 169.5 | 1893 | 599.5 | 617.5 | 40.13 |
| Sample 7 | 331 | 2322 | 666.5 | 836.5 | 40.33 |
| Sample 8 | 776 | 2279 | 476 | 964.5 | 25.46 |
| Sample 9 | 928 | 882.5 | 230 | 917 | 44.49 |
| Sample 10 | 730 | 707 | 346.5 | 449.5 | 17.65 |
| Sample 11 | 871.5 | 692.5 | 269.5 | 584.5 | 42.79 |
| Sample 12 | 829 | 1384 | 206 | 682.5 | 21.5 |
| Sample 13 | 326 | 3303 | 322 | 644 | 17.99 |
| Sample 14 | 161 | 3539.5 | 190 | 460 | 20.84 |
| Sample 15 | 170 | 3897.5 | 367.5 | 312 | 25.84 |
| Sample 16 | 166 | 1110 | 283 | 261.5 | 18.59 |
| **mean** | **464.69** | **1768.8** | **380.78** | **735.41** | **28.02** |
| **median** | **346.75** | **1231** | **349** | **706.5** | **25.65** |
