# Supplementary material for: About the influence of environmental factors on the persistence of DNA — a long-term study
Source: Int J Legal Med. 2022 Feb 23;136(3):687–93. doi: 10.1007/s00414-022-02800-6 (PMC9005405; doi:10.1007/s00414-022-02800-6)
Supplement: Supplementary file 5 — Supplementary file5 (DOCX 12.6 KB) [file 414_2022_2800_MOESM5_ESM.docx]

**Table S4: Distribution of the amount of complete profiles with regard to cell origin and season**

|  | Blood samples  (n = 240)* | Saliva samples  (n = 240)* | Epithelial samples  (n = 120)* |
| --- | --- | --- | --- |
| Complete profiles in summer (n = 155) | 76  ≙ 49 % of CP in summer  ≙ 63 % of summer blood samples | 62  ≙ 40 % of CP in summer  ≙ 52 % of summer saliva samples | 17  ≙ 11 % of CP in summer  ≙ 32 % of summer epithelial samples |
| Complete profiles in winter (n = 112) | 51  ≙ 46 % of CP in winter  ≙ 43 % of winter blood samples | 47  ≙ 42 % of CP in winter  ≙ 39 % of winter saliva samples | 14  ≙ 12 % of CP in winter  ≙ 27 % of winter epithelial samples |

* half of samples belong to the summer scenarios, the other half to the winter scenarios
